# Timing is everything: Clinical courses of Hunter syndrome associated with age at initiation of therapy in a sibling pair

**DOI:** 10.1016/j.ymgmr.2022.100845

**Published:** 2022-02-02

**Authors:** Nathan Grant, Young Bae Sohn, N. Matthew Ellinwood, Ericka Okenfuss, Bryce A. Mendelsohn, Leslie E. Lynch, Elizabeth A. Braunlin, Paul R. Harmatz, Julie B. Eisengart

**Affiliations:** a Department of Pediatrics, University of Minnesota, Minneapolis, MN, USA; b Department of Medical Genetics, Ajou University Hospital, Ajou University School of Medicine, Suwon, Republic of Korea; c National MPS Society, Durham, NC, USA; d Kaiser Permanente, Oakland, CA, USA; e UCSF Benioff Children's Hospital Oakland, Oakland, CA, USA

**Keywords:** Newborn screening, Mucopolysaccharidosis type II, Hunter syndrome, Enzyme replacement therapy, Early intervention, Sibling study, ABR, Auditory brainstem response, CNS, central nervous system;, DAS-II, Differential Ability Scales, Second Edition;, ERT, enzyme replacement therapy;, GAG, glycosaminoglycan;, HCT, hematopoietic cell transplantation;, IDS, iduronate-2-sulphatase;, IT, intrathecal;, MPS, mucopolysaccharidosis;, MPS II, mucopolysaccharidosis type II, Hunter syndrome;, MRI, magnetic resonance imaging;, NBS, newborn screening;, RUSP, Recommended Uniform Screening Panel

## Abstract

Hunter syndrome, or mucopolysaccharidosis (MPS) II, is a rare lysosomal disorder characterized by progressive, multi-system disease. As most symptoms cannot be reversed once established, early detection and treatment prior to the onset of clinical symptoms are critical. However, it is difficult to identify affected individuals early in disease, and therefore the long-term outcomes of initiating treatment during this optimal time period are incompletely described. We report long-term clinical outcomes of treatment when initiated prior to obvious clinical signs by comparing the courses of two siblings with neuronopathic Hunter syndrome (c.1504 T > G[p.W502G]), one who was diagnosed due to clinical disease (Sibling-O, age 3.7 years) and the other who was diagnosed before disease was evident (Sibling-Y, age 12 months), due to his older sibling's findings. The brothers began enzyme replacement therapy within a month of diagnosis. Around the age of 5 years, Sibling-O had a cognitive measurement score in the impaired range of <55 (average range 85–115), whereas Sibling-Y at this age received a score of 91. Sibling-O has never achieved toilet training and needs direct assistance with toileting, dressing, and washing, while Sibling-Y is fully toilet-trained and requires less assistance with daily activities. Both siblings have demonstrated sensory-seeking behaviors, hyperactivity, impulsivity, and sleep difficulties; however, Sibling-O demonstrates physical behaviors that his brother does not, namely biting, pushing, and frequent elopement. Since the time of diagnosis, Sibling-O has had significant joint contractures and a steady deterioration in mobility leading to the need for an adaptive stroller at age 11, while Sibling-Y at age 10.5 could hike more than 6 miles without assistance. After nearly a decade of therapy, there were more severe and life-limiting disease manifestations for Sibling-O; data from caregiver interview indicated substantial differences in Quality of Life for the child and the family, dependent on timing of ERT. The findings from this sibling pair provide evidence of superior somatic and neurocognitive outcomes associated with presymptomatic treatment of Hunter syndrome, aligned with current considerations for newborn screening.

## 1 Introduction

Hunter syndrome, or mucopolysaccharidosis type II (MPS II, OMIM #309900), is a progressive disorder that affects all body systems, leading to increasing multi-organ dysfunction and shortened lifespan, as well as a worsening quality of life for the affected person and family [1], [2], [3], [4]. This X-linked lysosomal disorder is associated with deficient activity of the enzyme iduronate-2-sulphatase (IDS, EC 3.1.6.13), which is required for complete break-down of the glycosaminoglycans (GAGs) heparan and dermatan sulfates. Continuously accumulating GAGs trigger pathogenic cascades that lead to progressive and generally irreversible clinical disease. There is considerable variability in the Hunter syndrome phenotype, in terms of both somatic and neurologic manifestations. The spectrum of functional neurologic impacts ranges from the more common neuronopathic form, which involves neurodegeneration manifesting as developmental regression and intense neurobehavioral challenges, to the non-neuronopathic form, which involves generally average intelligence and comportment [4], [5], [6], [7], [8]. In recent years, the non-neuronopathic form has been found to have impairments in attention and visual-motor skills [9], indicating this classification is not completely free of neurologic deficits as the name suggests. Severity of neurologic involvement does not predict severity of somatic disease, which is always present regardless of phenotype and can be severe even in neurocognitively unaffected individuals [9], [10], [11]. The most common somatic signs include airway disease, dysostosis multiplex, joint stiffness and severely restricted range of motion, carpal tunnel syndrome, hearing loss, cardiac involvement, hepatosplenomegaly, and facial coarsening [12].

Clinical disease in Hunter syndrome steadily progresses, and as with other MPS disorders, many symptoms may be irreversible once evident [13]. Thus, it is critical to initiate treatment prior to clinical signs. In the US, the only FDA-approved treatment is intravenous infusion of recombinant IDS (idursulfase) which works to replace the deficient enzyme (i.e., enzyme replacement therapy, ERT). However, Hunter syndrome is extremely challenging to identify early in disease, as affected infants and toddlers appear physically normal, and those with the neuronopathic phenotype gain early skills before disease processes overcome development, causing regression [1], [2], [11], [14], [15]. Early detection is a critical problem. The technology is in place to identify Hunter syndrome with newborn screening (NBS) and active screening programs exist in Taiwan, Illinois and Missouri [16], [17], [18]. The advent of NBS technology, combined with the 2006 FDA approval of ERT to treat Hunter syndrome, position this disorder to be considered by the U.S. Secretary of Health and Human Services for addition to the Recommended Uniform Screening Panel (RUSP). One criterion for addition to the RUSP panel is urgency of therapy in the newborn period, but there is presently a paucity of published data documenting the long-term controlled outcomes of early versus later initiation of treatment [19]; this is partly because it has been difficult to recognize and therefore treat children with Hunter syndrome prior to clinical signs. Early disease detection prompted by diagnosis in an elder sibling proband is an important approach to understanding this critical question, as sibship pathological variants are the same, phenotypes are largely similar, and there are reduced, although not eliminated, differences in genetic background and environmental factors that might influence outcomes [20], [21]. The present study provides a critical opportunity to describe long-term clinical outcomes of treatment when initiated prior to obvious clinical signs by comparing the courses of two siblings with neuronopathic Hunter syndrome, one who was diagnosed due to clinical disease and the other who was diagnosed before disease was evident, due to his older sibling's findings.

## 2 Patients and methods

### 2.1 Patients

The two brothers have the neuronopathic phenotype of Hunter syndrome, carrying c.1504 T > G (p.W502G) hemizygous mutation in *IDS* along with undetectable iduronate-2-sulfatase enzyme activity. The parents are non-consanguineous Chinese individuals with a negative familial history for inherited diseases. The older brother (Sibling-O) was diagnosed at 3 years, 8 months old, prompting evaluation in the younger brother (Sibling-Y), who was thus diagnosed at 12 months old.

#### 2.1.1 Treatment histories

Both brothers began receiving weekly intravenous ERT the month following diagnosis, i.e., at ages 3 years, 9 months and 13 months, respectively. They were later enrolled in a clinical trial of intrathecal (IT) idursulfase (NCT02055118) and withdrew: Sibling-O participated in the clinical trial from 6 years, 6 months old to 10 years, 2 months old, while Sibling-Y participated from 5 years, 6 months old to 9 years, 9 months old. Sibling-Y began another clinical trial of CNS-penetrant ERT (NCT04251026) at 10 years, 2 months old. Sibling-O stopped all ERT at age 11 years, 8 months due to progression of neurodegeneration, and has received palliative care.

#### 2.1.2 Birth histories and diagnosis

Sibling-O was born at 40 weeks of gestation with a birth weight of 3220 g following an uneventful pregnancy. Developmental milestones within the first year of life were met in normal timeframes, and he started to walk independently at one year. Progressive joint stiffness, clumsiness in fine motor skills, and delayed language development prompted increasing concern. By 3 years, 8 months, mucopolysaccharidosis was suspected in light of developmental delay, macrocephaly, mildly coarse facial appearance, mild hepatosplenomegaly, joint contractures and skeletal deformities. Elevated urinary GAG measured using 1,9-dimethyl-methylene blue (DMB) incorporation and spectrophotometry, undetectable IDS enzyme activity, and identification of a causative IDS allele confirmed the diagnosis of Hunter syndrome.

Sibling-Y was born at 40 weeks of gestation with a birth weight of 3180 g following an uneventful pregnancy. Upon diagnosis, Sibling-Y had no apparent clinical symptoms of Hunter syndrome except macrocephaly. Developmental milestones were in the normal range at diagnosis.

### 2.2 Methods

A multi-method approach was used to assemble outcome data spanning approximately a decade: 1) Retrospective review of medical chart data provided by the parents from multi-disciplinary clinical records since birth; 2) Retrospective review of symptom logs recorded by the parents; 3) Virtual semi-structured interviews with the siblings' father with focus on the boys' lived experiences with Hunter syndrome including neurobehavioral manifestations, caregiver experiences, and overall quality of life. Within Method 1, cardiac echo reports were reviewed from the time of diagnosis and most recently; echo images were not available for review for this manuscript. This study (protocol STUDY00014051) was reviewed by the University of Minnesota Institutional Review Board, which determined it was not research requiring oversight of more than 3 human subjects as defined by DHHS and FDA regulations.

## 3 Results

### 3.1 Central nervous system

#### 3.1.1 Structural

On brain and cervical MRI, the structural differences between the siblings were minimal, with stability of findings per the most recent MRI exams (performed around the age of 10). At the time of diagnosis both siblings had macrocephaly (+2.40 and + 1.83 SDs for Sibling-O and Sibling-Y, respectively), mild ventriculomegaly, and enlarged perivascular spaces in the corpus callosum. Neither sibling had shown clinical evidence of any of the following: hydrocephalus, increased intracranial pressure, seizures, cervical cord compression, myelomalacia, nor carpal tunnel syndrome. At age six, Sibling-O showed mild changes of dysostosis within the cervical spine. At age five, Sibling-Y was found to have mild cervical spinal stenosis.

#### 3.1.2 Neurocognitive

**Fig. 1:**
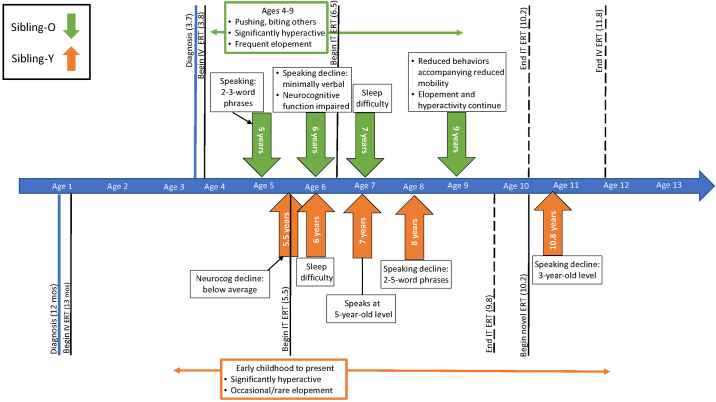
Neurocognitive and neurobehavioral symptom progression and systemic treatments. This timeline depicts siblings' ages of key functional symptom onset or change, as identified on medical exam or by caregiver observation. Timing of initiating therapies (and cessation, when applicable) is also represented. While symptom onset and/or change is evident across both boys' lives, the severity of symptoms and functional impairments is greater for Sibling-O (green).

Key neurocognitive and neurobehavioral outcomes are presented according to timing and type of treatment, i.e., before standard intravenous ERT, before enrollment in IT idursulfase, and before Sibling-Y began the clinical trial of CNS-penetrant ERT (Fig. 1).

Substantial differences in neurocognitive functioning were quantified when the boys each were five years old, when they underwent neurocognitive testing to determine eligibility for the clinical trial of IT idursulfase. For this trial, an inclusion criterion was a neurocognitive score measuring between 55 and 85 (population mean = 100, SD = 15; average range is 85–115) on the Differential Ability Scales, Second Edition (DAS-II) [22]. Both siblings were excluded from the trial for functioning that measured outside of the eligibility range: below the range for Sibling-O, but above the range for Sibling-Y. Scores from trial screening were provided by the trial sponsor (Takeda Pharmaceutical Company Ltd) and are included with permission. Specifically, at 5.5 years old, Sibling-O received a total DAS-II score of 46. By contrast, at five years old Sibling-Y received a score of 91. Retest of Sibling-Y about three months later yielded a score of 87, but after another three months, his score was 79, thus rendering him eligible to enroll in the trial. Sibling-O was regularly retested and consistently scored below 55 for the next year, but at 6.5 years old, he earned a score of 59, allowing him to enroll. Parent interview indicated that his behavioral symptoms were less challenging that day, which they speculated to be the factor that enabled him to earn more points. Post-enrollment neurocognitive testing results for either boy are not available as this is an ongoing trial.

Qualitatively, parent interview data indicate that Sibling-Y continues to remain engaged with activities requiring greater neurocognitive skills, such as understanding stories and movies and assembling 200–300-piece puzzles, whereas Sibling-O does not.

With respect to expressive language, at age five, Sibling-O communicated primarily via 2–3 word phrases with vocabulary estimated at approximately 50 words. He remained verbal until age six, after which he has been minimally verbal, occasionally saying single words. Sibling-Y has always been more verbal than Sibling-O, per parents. He showed concerns with language at age seven, at which time a speech/language pathologist estimated his communication to be a five- or six-year-old level. At age eight an assessment indicated speech phrases were 2–5 words. Sibling-Y now (age 10.7) communicates at the level of a three-year-old; he can understand more than he can speak.

#### 3.1.3 Neurobehavioral

Between ages four to nine, Sibling-O engaged in physical behaviors including forcefully pushing others and biting. His parents said these behaviors were primarily efforts to obtain sensory feedback and denied that they were aggression-based, citing his overall positive outlook and happy demeanor, even when showing the behaviors. Frequency decreased with the use of medications (including various trials of antipsychotics, hypotensive agents and psychostimulants), and as his mobility further deteriorated around the age of nine. An ongoing concern is that Sibling-O frequently elopes and demonstrates a diminished sense of fear. Parents and other caregivers must pay close attention to Sibling-O in public to ensure he does not run away. Sibling-O requires a one-on-one aide at school for safety and learning needs.

Sibling-Y has not forcefully pushed or bitten others. He has been less prone to elope and generally stays near his caregivers. Sibling-Y requires a one-on-one aide at school for learning and behavioral support needs.

Both siblings have been hyperactive and impulsive since early childhood. Around the age of seven, both siblings began taking medications for hyperactivity which have been helpful. They are both described by their parents as generally very happy and playful children.

#### 3.1.4 Sleep

Difficulties falling and staying asleep began at ages six and seven for Sibling-Y and Sibling-O, respectively. Sibling-O has more frequent sleep difficulty. Both siblings began medications to help with sleep at age seven.

### 3.2 Sensory

#### 3.2.1 Vision

Sibling-Y was diagnosed with mild myopia at age four (left eye −2.00; right eye −1.00) and was prescribed glasses, but he does not often wear them; his eyesight slightly changed at age seven (left eye unchanged; right eye −2.00) and has since remained stable. Sibling-O has shown no signs of visual impairment.

#### 3.2.2 Hearing

Auditory brainstem response (ABR) evaluation indicated normal hearing thresholds bilaterally (20–25 dB) for Sibling-O at age eight, and hearing has remained stable. ABR for Sibling-Y showed bilateral sensorineural hearing loss (50–60 dB of thresholds) at age four and he has hearing aids. Neither sibling has had recurring ear infections.

### 3.3 Dental

Tightness in Sibling-O's temporomandibular joint creates difficulty assisting him with brushing and flossing. By age five, he developed several cavities in his primary teeth, which were filled. He requires specialty dental care with papoose restriction due to neurobehavioral symptoms.

Sibling-Y's temporomandibular joint is not restricted, improving access for brushing and flossing, and he has not developed any cavities. Sibling-Y tolerates dental exams and sees a family dentist with adult assistance.

### 3.4 Swallowing

Sibling-O started to experience difficulties with swallowing at age 13. Sibling-Y has no swallowing difficulty.

### 3.5 Pulmonary

Neither sibling has shown significant airway obstruction, sleep apnea, snoring, or recurrent respiratory infections.

### 3.6 Cardiac

Longitudinal cardiac echo findings are presented in Table 1. There was a marked difference in valve appearance between Sibling-O and Sibling-Y on initial cardiac echoes. Sibling-O had mild thickening of both mitral and aortic valves while Sibling-Y had no thickening. Neither brother had valve regurgitation at initial echo. By about 10 years of age, Sibling-O had unmistakable thickening of both mitral and aortic valves and had developed mild aortic regurgitation. Sibling-Y had also developed aortic valve thickening and doming but there was no aortic insufficiency. Ventricular function (shortening fraction) was normal for both brothers at all time points where it was available. Neither sibling is prescribed any cardiac medications at this time.

**Table 1:** Summary of cardiac echo findings over time.

Cardiac Parameter	Sibling-O Age (years:months) – Assessment	Sibling-Y Age (years:months) – Assessment
Mitral valve thickening	• 3:9 – Mild • 10:1 – Thickened	• 1:1 – None • 9:8 – Mild
Mitral valve regurgitation	• 3:9 – None • 10:1 – Trace	• 1:1 – None • 9:8 – Trivial
Aortic valve thickening	• 3:9 – Mild • 10:1 – Thickened	• 1:1 – None • 9:8 – Thickened-doming
Aortic regurgitation	• 3:9 – None • 10:1 – Mild	• 1:1 – None • 9:8 – None
Shortening fraction	• 3:9–45.9 • 10:1–42.1	• 1:1–47.42 • 9:8 – Missing (report said ‘normal’)

### 3.7 Gastrointestinal

At the time of diagnosis, Sibling-O had an enlarged liver and spleen (palpable two fingerbreadths below the costal margin), with an abdominal ultrasound revealing hepatosplenomegaly with homogeneous echogenicity. After six months of ERT, the liver and spleen were no longer palpable. Liver and spleen were not palpable at the time of Sibling-Y's diagnosis and have remained as such.

Neither sibling has had inguinal hernias, nor gastrointestinal complications such as frequent vomiting, diarrhea, or constipation.

### 3.8 Musculoskeletal

**Fig. 2:**
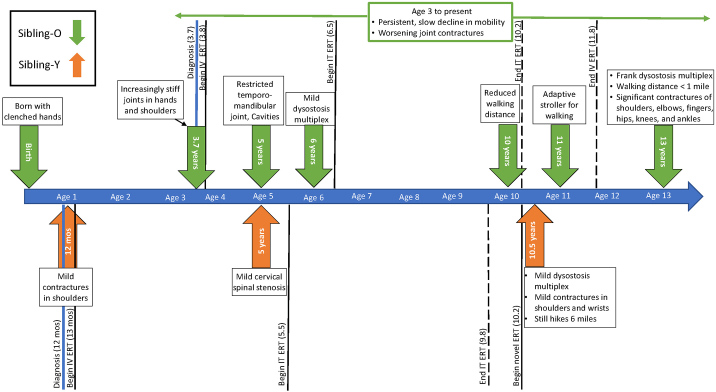
Timeline of musculoskeletal findings and systemic treatments. This timeline depicts siblings' ages of key musculoskeletal symptom onset or change, as identified on medical exam or by caregiver observation. Timing of initiating therapies (and cessation, when applicable) is also represented. While symptom onset and/or change is evident across both boys' lives, the severity of symptoms and functional impairments is greater for Sibling-O (green).

Key musculoskeletal findings are presented over time, also representing type of treatment, i.e., before standard intravenous ERT, before enrollment in IT idursulfase, and before Sibling-Y began the clinical trial of CNS-penetrant ERT (Fig. 2).

#### 3.8.1 Growth

**Fig. 3:**
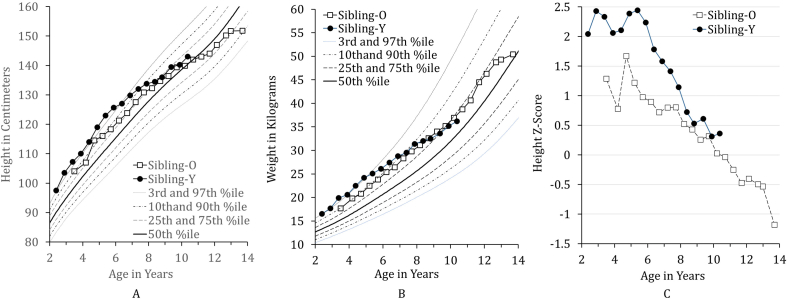
The siblings' growth charts for height (A), weight (B), and height *Z*-score (C). Height growth curves for both boys overlapped until age 10, after which Sibling-O showed more deceleration of growth. Weight curves overlapped until age 8, after which Sibling-Y decreased in velocity of weight gain.

Both siblings reached the 97th percentile for height and weight by age 3. Their height growth curves overlapped until the age of 10, with heights at the 97th percentile until six years of age and reducing to the 90th percentile by 7.5 years of age (Fig. 3A). The growth velocities of the siblings diverged thereafter (height *Z*-scores are shown in Fig. 3C):

Sibling-O: By 10.5 years, height measured at the 50th percentile with annual growth velocities ranging from approximately 4.7 cm/year to 2.6 cm/year from age 7.5 to 10.5. A pubertal growth spurt (8 cm gain across 1 year, i.e., between 11 and 12 years old) was followed by rapid decrease in growth velocity after age 12 years, with most recent estimated growth velocity to be 0.2 cm/year at 13.5. Height at 13.5 years (most recent measurement) was 151.8 cm (25th percentile) and he was thought to be approaching his final adult height based on bone age of 14 years.

Sibling-Y: By 9.5 years, height was at the 75th percentile, with annual growth velocities ranging from approximately 1.5 cm/year to 5.0 cm/year from age 7.5 to 9.5. At the most recent measurement (age 10.5 years), determined to be prepubertal, height was 143 cm (50-75th percentile); growth velocity at 9.5 was 3.5 cm/year.

Both siblings' weights were 97 percentile until 5 years old. Up to 8 years of age, the weight curves of both siblings showed a similar pattern (Fig. 3B).

#### 3.8.2 Joint

**Fig. 4:**
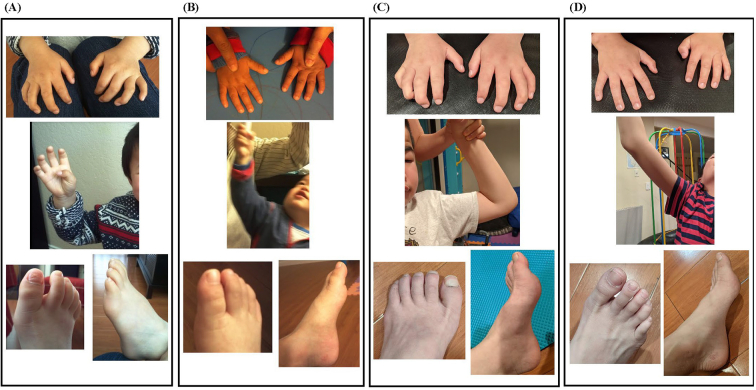
Comparison of joint progression over about a decade. Photographs of joint contractures of the hands, restricted shoulder range of motion, and pes cavus deformity for each sibling. Sibling-O's joint disease is evident at age 4.3 years, i.e., about 6 months on ERT (A), including significantly restricted shoulder ROM preventing reach much above the browline. By contrast, Sibling-Y's joint disease is more attenuated at age 1.5 years, i.e., also about 6 months on ERT (B), including the ability to reach above the head. After around a decade of therapy, Sibling-O at age 13 years (C) showed persistent contractures despite ERT. In comparison, Sibling-Y's joint disease at age 11 years (D) showed no significant contractures except mild limitations in shoulders and hands. Photographs were provided by the parents and used with permission. ERT, enzyme replacement therapy.

Sibling-O was born with clenched hands, which took several weeks of physical therapy to release. During early childhood, upper extremity joints including shoulders, wrists, and fingers became more contracted and stiffened, leading to clumsiness in fine motor skills. He started to walk independently at one year old, with frequent toe walking. Established joint contractures remained stable or progressed over 10 years of ERT (Fig. 4A and C), resulting in restricted mobility which has followed a constant slow decline since diagnosis. He has displayed more difficulties in walking in general. By age 10, independent walking distance shortened. By age 11, he started using an adaptive stroller outside when tired or on slopes. At 13, contractures are significant in shoulders, elbows, fingers, hips, knees and ankles; walking distance is less than one mile.

Sibling-Y: There were mild contractures only in shoulders at the time of diagnosis and initiation of ERT (Fig. 4B). At 10.5 years, Sibling-Y could independently hike more than six miles with mild elevations and he had no functional limitations in using his joints despite mild contractures in shoulder and wrist joints (Fig. 4D). Sibling-Y was able to learn how to swim and bike, whereas his older brother was not.

#### 3.8.3 Skeletal

At the time of diagnosis, Sibling-O had dysostosis multiplex including mild odontoid hypoplasia, inferiorly beaking vertebrae, and rounded iliac wings while Sibling-Y showed milder bone deformities in vertebrae and iliac bones. On skeletal survey at 13, Sibling-O had skeletal deformities including beaking vertebral bodies without scoliosis or kyphosis, flattened femoral head with dysplastic acetabuli, genu valgum, and pes cavus. On skeletal survey at 10, Sibling-Y had similar skeletal deformities but with milder degree of severity.

### 3.9 Laboratory

**Fig. 5:**
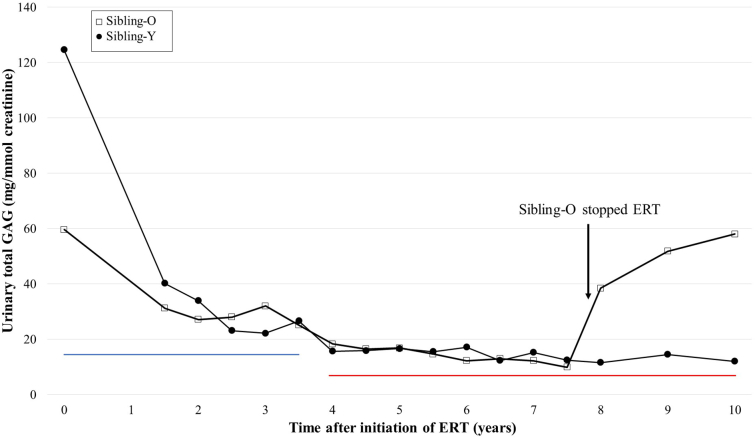
Changes in total urinary GAGs since initiation of ERT. Total urinary GAGs are plotted against years since initiating treatment with ERT. The urinary total GAGs were measured using 1,9-dimethyl-methylene blue (DMB) incorporation and spectrophotometry. The reference ranges were 0–16 mg/mmol Cr during the period of 0–3.5 years after initiation of ERT (blue line) and 0–6.5 mg/mmol Cr after 4 years (red line). Due to data availability, the first urinary GAG measurement for Sibling-O was obtained approximately 2 weeks after initiating ERT, and the first urinary GAG measurement for Sibling-Y was obtained before initiating ERT. After initiating ERT, urinary GAGs decreased in both siblings over a period of approximately 7 years. Around 11.7 years of age, Sibling-O experienced an increase in urinary GAGs after withdrawing from ERT and transitioning to palliative care due to progression of neurodegeneration. GAGs, glycosaminoglycans; ERT, enzyme replacement therapy.

At diagnosis, urinary total GAG was abnormally high in both siblings. Urinary GAG levels were reduced early in the course of IV ERT and remained stable during ERT. Sibling-O's urinary GAG levels were re-elevated after he stopped ERT (Fig. 5).

Sibling-O had measurable anti-IDS IgG antibodies 3 months after the initiation of ERT, which were undetectable after 2 years of ERT. Sibling-Y has not developed anti-IDS antibodies. After approximately 10 years on ERT, neither sibling has IDS-neutralizing antibodies or anti-IDS IgE antibodies.

### 3.10 Caregiving

#### 3.10.1 Care needs

Both siblings need help with daily living activities, although to varying degrees of required caregiver support, due to differences in mobility, neurocognitive function, and neurobehavioral symptoms.

Sibling-O has always required direct assistance with daily activities and tasks, such as washing and dressing. He has never been toilet trained, has always worn diapers, and requires assistance with toileting.

Sibling-Y can wash and dress himself with supervision, and he requires much less assistance than his brother. Sibling-Y is toilet trained.

#### 3.10.2 Family's perception of caregiving and quality of life

The siblings receive care primarily from their parents and sometimes from their grandparents. The parents reported that it can be difficult to provide care due to the siblings' hyperactivity, impulsivity, sensory-seeking behaviors, and sleep difficulties. The siblings were described as “keeping the adults in the house very busy.” However, over the past two years, the parents have experienced less stress providing care for Sibling-O due to his reduced mobility. Nevertheless, Sibling-O still continues to require significant attention and care, particularly with toileting which necessitates physical maneuvering, calming, and support from multiple adults.

The family's perception of quality of life for the children is that Sibling-Y's is superior, as his physical and mental capabilities offer him “a better chance of enjoying life.” Sibling-Y swims, bikes, and enjoys the outdoors more, can assemble 200–300 piece puzzles, and understands movies and stories for entertainment. He is able to enjoy independent movement and some independence in life activities. Sibling-O is much more restricted in his access to pleasurable activity, independent movement, and independence in life activities, thus limiting his free volition to fulfill his needs or desires on his own.

## 4 Discussion

We have chronicled the long-term outcomes, in system-by-system fashion, for a pair of siblings who began ERT for neuronopathic Hunter syndrome at different ages, because the diagnosis from clinical disease in the elder 3.7-year-old brother prompted diagnosis in the 12-month-old brother, who was minimally symptomatic. After nearly a decade of therapy, there were more severe and life-limiting disease manifestations for the elder-treated sibling (Sibling-O) in terms of skeletal/joint disease (with related limitations to mobility and basic dental care), and neurocognitive and neurobehavioral function. These differences in disease progression were felt by the parents to have a profound effect on the boys' quality of life, creating disparate experiences for the two boys, tied to the lag to starting ERT. The younger-treated brother, Sibling-Y, has “a better chance of enjoying life” due to his ability to engage in many classic childhood diversions such as biking, swimming, playing with puzzles, and understanding movies and stories, and to complete many tasks of daily living without the need for full hands-on adult assistance. By contrast, the elder-treated brother, Sibling-O, experiences disease-related barriers to all of these diversions as well as basic tasks of daily living due to his severely limited mobility and comprehension.

Findings of fewer and less severe disease manifestations in Sibling-Y align with a recent report that used statistical models to assess and to predict outcomes of ERT in patients from the Hunter Outcome Survey patient registry (NCT03292887); specifically, predicted outcomes after 5–8 years of ERT were more favorable across all clinical parameters for patients who began ERT before age 18 months [23]. Prior to this report, a Delphi consensus recommended presymptomatic ERT for neuronopathic MPS II [24]. Further, two other MPS II studies report superior treatment response for a presymptomatically diagnosed sibling compared to a clinically diagnosed older sibling. One such case study reported 32-month outcomes of standard ERT in a sibling pair who began treatment at 3 years old and 4 months old, respectively: The younger-treated sibling was generally spared most of the somatic complications of Hunter syndrome seen when his older brother had been his same age, including joint contractures, and overall, dysostosis multiplex and neurocognitive impairment were much milder in the younger child [25]. A more recent sibling case study involved prenatal diagnosis of one child following the diagnosis in a 2-year-old sibling, with standard ERT beginning at 1 month old and 2 years old, respectively [26]. The younger child was transitioned to a blood-brain barrier penetrating ERT at age 1 year 11 months whereas the elder remained on standard ERT. The two-year follow-up data suggest the younger sibling had not developed any disease symptoms and maintained an average neurocognitive developmental trajectory, whereas the elder sibling's course involved several systemic manifestations of disease and neurocognitive impairments. In both of these case reports, the authors called for longer-term examination of outcomes.

Skeletal and joint disease are pervasive and difficult to address manifestations of Hunter syndrome, regardless of phenotype. Improvements in these areas have been assumed to improve function and quality of life in MPS II [19], and the present data support these assumptions. Prevention of skeletal and connective tissue involvement of a patient with MPS I treated with ERT from birth [27] raises the possibility that the superior outcomes for Sibling-Y may have been even better, were ERT started before age 13 months.

Cardiac valvulopathy was recently found to have a higher incidence over a 10-year follow-up period than in previous reports [11]. In line with those findings, Sibling-O showed mild cardiac valve thickening at 3 years 9 months, but by 10 years of age developed unmistakable valve thickening of both valves and mild aortic regurgitation. By contrast, Sibling-Y showed no valve thickening at 13 months of age but mild thickening of the aortic valve, with neither regurgitation nor stenosis of the valve, over the ensuing 10 years. The cardiac findings seen in these brothers are subtle but lend support to the importance of early treatment in delaying the onset of, and possibly attenuating, cardiac valvulopathy in MPS II. Indeed, the only therapeutic approach to fully prevent difficult-to-address pathologies, such as cardiac disease, in large animal MPS models involved therapy from birth, such as in the canine model in MPS I [28]. With first echo and treatment at 13 months of age, Sibling-Y was already ‘old' by standards for MPS I, which was added to the RUSP in 2016. Thanks to NBS, most children with severe MPS I would already have begun ERT within weeks of birth, and hematopoietic cell transplantation (HCT) within the first 6–9 months of life, if not earlier.

Therapy from birth would be enabled by newborn screening for MPS II, which is under consideration for addition to the RUSP. MPS I was added to the RUSP in large part due to the evidence demonstrating neurocognitive benefit of early HCT for severe MPS I [29]. The present report suggests a functional benefit for MPS II, because when each brother was around 5 years old, Sibling-Y measured too neurocognitively high, and Sibling-O measured too low, to meet eligibility criteria for enrollment in a clinical trial (NCT02055118). Neurobehavioral impairments also differed, with the elder-treated brother showing substantially more physical behaviors (e.g. forcefully pushing others and biting) than his younger brother. Parent explanation of these behaviors as non-aggressive but rather sensory-seeking is consistent with a recent report of the multiple meanings behind the neurobehavioral features of this condition [4]. Both brothers, but to a more significant degree Sibling-O, display elopement behaviors, which are a cause of premature death due to drowning, traffic accident, and injury in pediatric populations with neurodevelopmental differences such as autism [[30], [31]].

Taken together, the present study offers unexpected data suggesting neurocognitive and neurobehavioral benefit associated with earlier initiation of ERT for neuronopathic MPS II. Our findings may be informed by recent animal model findings that early initiation of high-dose ERT decreases brain GAG accumulation, ameliorates brain tissue damage, and improves behavior, in mice with MPS II [[32], [33]]. Crucial to this work was not just the high dose of the ERT but also the early timing. Although it is believed that standard intravenous ERT does not appreciably cross the blood-brain barrier, reports suggest that a small fraction of enzyme is able to enter the CNS [[34], [35], [36]], with hypothesized mechanisms that include pinocytosis [37], extracellular pathways [38], more efficient uptake via mannose-6-phosphate receptors [32], or other presently unrecognized ones. The entry of enzyme into the CNS is a critical focus of therapeutic development, as recent reviews have described therapeutics in development or in trial, such as brain-penetrating ERT and gene therapies [[39], [40], [41], [42], [43]]. HCT is the standard of care for MPS I but has had varying outcomes for MPS II [44], with concerns about a lack of controlled clinical studies of this approach for MPS II [1]; however, there is some evidence that earlier treatment is key [45].

Better CNS functional status with early ERT has been seen in MPS I, with speculation that general feelings of wellness and increased physical flexibility enabled more opportunities for learning and absorbing information without the burdensome distraction of intense pain, joint disease, disrupted sleep, and/or other disease symptoms [[46], [47]]. There is evidence that somatic symptoms can have more direct effects on neurocognitive function. For example sleep apnea, a frequent disease manifestation in Hunter syndrome [[1], [48]], has been linked to a host of neurocognitive insults in the general population, including impairments in intellectual skills, academic performance, attention, memory, behavior, mood, and self-regulation [[49], [50]]. Sleep apnea has also specifically been named as a contributor to the neurobehavioral complications and learning challenges of neuronopathic MPS II [[1], [4]].

Regardless of the potential explanations for differences in neurocognitive and neurobehavioral signs between these siblings, a significant problem with MPS II is that the prediction of phenotype is challenging for a larger segment of the MPS II population than for MPS I. In MPS II, phenotype based on genotype can be particularly unreliable, except in cases of previously characterized disease-causing alleles [51]. However, a lack of neurocognitive phenotype prediction does not actually create a significant quandary with respect to initiating ERT, as this therapy is approved to treat somatic disease, and further, the present study demonstrated benefits distinct and independent from the benefits for neurocognitive and neurobehavioral function. Specifically, the differences in skeletal/joint disease (Fig. 3) and mobility have determined the types of activities that the boys may engage in, with far greater limits for the elder-treated boy. While the neurocognitive and neurobehavioral symptoms undoubtedly have a role in reducing the boys' independence, it is not neurodegeneration underlying Sibling-O's need for an adaptive stroller, but rather severe, multiple joint contractures and skeletal disease. Skeletal and other somatic disease manifestations are present regardless of phenotype and can be severe even in individuals with minimal neurocognitive effects [9], [10], [11], which is a critical point to consider when concerned about phenotype prediction.

While caregiver and family burden is incompletely characterized in MPS disorders [[4], [6], [7], [52], [53], [54]], the present report illustrates caregiver physical strain associated with the joint and mobility limitations experienced by Sibling-O, whose physical support needs require intensive and continual caregiver assistance throughout the day. This physical assistance for a growing child who loses mobility has been reported as a serious factor in caregiver burden in another neuronopathic MPS, Sanfilippo syndrome [[53], [54]]. Thus the physical complications may be implicated in “spillover effects” on caregiver health, as the physical strain of caregiving is considerable, and there have been calls to conceptualize “health as a family affair” [55]. Information on the caregiver experience has been recognized by regulatory bodies as an important source of information on disease progression and response to treatment [56].

One limitation of the present report is that although the two siblings carry the same variant, their genetic background is not identical which could influence some of the results. However, previous groups have reported less significant or less common impact of genetic background within sibships [20], [21], suggesting a superior comparison than unrelated children in different households. Further, recent work suggests importance of genetic variant as a predictor of course of neurocognitive decline [57], which may strengthen the likelihood that the present brothers' courses would have been similar. As another limitation, the siblings in this study have participated in different clinical trials. Unchanged treatment with only FDA-approved therapy may have afforded a purer analysis of disease change over time, and could reduce doubt that some of Sibling-Y's benefit was attributable to novel therapy rather than early intervention. However, benefit of pre-symptomatic treatment was already evident before enrollment in the first clinical trial, as seen by striking differences in joint disease (Fig. 3) and in neurocognitive function.

With a number of novel therapies targeting CNS function currently approved outside the United States [58], in trial (NCT04571970, NCT03566043, and NCT04251026), or in development, it is likely that the treatment picture for MPS II will soon be changing, but decisions about therapy-from-birth are still meaningfully and actionably informed by the current report and others [25], [26]. However, it is important to note that while newborn screening is complex to approve and implement [[59], [60]], and may be a first step of many in improving outcomes, there has been recent attention to the surge in therapies' outpacing the additions of disorders to NBS panels [60]. In the case of MPS II, NBS is a worthy, even if complicated, step: Earliest intervention will provide the opportunity for improved scientific examination of therapeutic approaches, potentially clarifying the best possible outcomes for the child and family.

## 5 Conclusions

This report addresses previously posed questions about whether the differential benefits of earlier initiation of therapy remain in the long-term. The data suggest persistently reduced severity and occurrence of symptoms for the younger treated sibling than the older, over a decade of treatment. An important contribution of the current study is the parent perspective on the lived experience of the children and the caregivers, which revealed significant benefits to the quality of life for the child and separately for the family, afforded by earlier initiation of therapy. Findings strengthen the argument for earlier treatment that would be enabled by newborn screening.

## Funding

Supported by the Neurodevelopmental Program in Rare Disease, 10.13039/100007249University of Minnesota.

## References

[1] Muenzer J., Beck M., Eng C., Escolar M., Giugliani R., Guffon N., Harmatz P., Kamin W., Kampmann C., Koseoglu S. (2009). Multidisc. Manag. Hunter Syndr. Pediatr..

[2] Neufeld E., Muenzer J. (2001). The Metabolic and Molecular Bases of Inherited Disease.

[3] Jones S., Almassy Z., Beck M., Burt K., Clarke J., Giugliani R., Hendriksz C., Kroepfl T., Lavery L., Lin S.-P. (2009). Mortality and cause of death in mucopolysaccharidosis type II—a historical review based on data from the hunter outcome survey (HOS). J. Inherit. Metab. Dis..

[4] Eisengart J., King K., Shapiro E., Whitley C., Muenzer J. (2020). The nature and impact of neurobehavioral symptoms in neuronopathic Hunter syndrome. Mol. Genet. Metab. Rep..

[5] Shapiro E.G., Jones S.A., Escolar M.L. (2017). Developmental and behavioral aspects of mucopolysaccharidoses with brain manifestations—neurological signs and symptoms. Mol. Genet. Metabolism.

[6] Grant N. (2018). Sibling and family caregivers. BMJ.

[7] Grant N., Von Handorf R., Karaa A., Skotko B.G. (2021). The experiences and support needs of siblings of people with mucopolysaccharidosis. Am. J. Med. Genet. A.

[8] Roberts J., Stewart C., Kearney S. (2016). Management of the behavioural manifestations of hunter syndrome. Br. J. Nurs..

[9] Yund B., Rudser K., Ahmed A., Kovac V., Nestrasil I., Raiman J., Mamak E., Harmatz P., Steiner R., Lau H., Vekaria P., Wozniak J.R., Lim K.O., Delaney K., Whitley C., Shapiro E. (2015). Cognitive, medical, and neuroimaging characteristics of attenuated mucopolysaccharidosis type II. Mol. Genet. Metabolism.

[10] Shapiro E.G., Rudser K., Ahmed A., Steiner R.D., Delaney K.A., Yund B., King K., Kunin-Batson A., Eisengart J., Whitley C.B. (2016). A longitudinal study of emotional adjustment, quality of life and adaptive function in attenuated MPS II. Mol. Genet. Metab. Reports.

[11] Broomfield A., Davison J., Roberts J., Stewart C., Hensman P., Beesley C., Tylee K., Rust S., Schwahn B., Jameson E. (2020). Ten years of enzyme replacement therapy in paediatric onset mucopolysaccharidosis II in England. Mol. Genet. Metabolism.

[12] Burton B.K., Giugliani R. (2012). Diagnosing hunter syndrome in pediatric practice: practical considerations and common pitfalls. Eur. J. Pediatr..

[13] Muenzer J. (2014). Early initiation of enzyme replacement therapy for the mucopolysaccharidoses. Mol. Genet. Metabolism.

[14] Holt J., Poe M.D., Escolar M.L. (2011). Early clinical markers of central nervous system involvement in mucopolysaccharidosis type II.

[15] Holt J.B., Poe M.D., Escolar M.L. (2011). Natural progression of neurological disease in mucopolysaccharidosis type II. Pediatrics.

[16] Chuang C.-K., Lee C.-L., Tu R.-Y., Lo Y.-T., Sisca F., Chang Y.-H., Liu M.-Y., Liu H.-Y., Chen H.-J., Kao S.-M. (2021). Nationwide Newborn Screening Program for Mucopolysaccharidoses in Taiwan and an Update of the “Gold Standard” Criteria Required to Make a Confirmatory Diagnosis Diagnostics.

[17] Burton B.K., Hickey R., Hitchins L. (2020). Newborn screening for mucopolysaccharidosis type II in Illinois: an update. Int. J. Neonat. Screening.

[18] Bilyeu H., Washburn J., Vermette L., Klug T. (2020). Validation and implementation of a highly sensitive and efficient newborn screening assay for mucopolysaccharidosis type II. Int. J. Neonat. Screening.

[19] Bradley L.A., Haddow H.R., Palomaki G.E. (2017). Treatment of mucopolysaccharidosis type II (Hunter syndrome): results from a systematic evidence review. Genet. Med..

[20] Ficicioglu C., Giugliani R., Harmatz P., Mendelsohn N.J., Jego V., Parini R. (2018). Intrafamilial variability in the clinical manifestations of mucopolysaccharidosis type II: data from the hunter outcome survey (HOS). Am. J. Med. Genet. A.

[21] Young I., Harper P., Archer I., Newcombe R. (1982). A clinical and genetic study of Hunter's syndrome. 1 heterogeneity. J. Med. Genet..

[22] Elliott C.D., Salerno J.D., Dumont R., Willis J.O. (2018). Contemporary Intellectual Assessment: Theories, Tests, and Issues.

[23] Muenzer J., Botha J., Harmatz P., Giugliani R., Kampmann C., Burton B.K. (2021). Evaluation of the long-term treatment effects of intravenous idursulfase in patients with mucopolysaccharidosis II (MPS II) using statistical modeling: data from the Hunter Outcome Survey (HOS). Orphanet J. Rare Diseases.

[24] McBride K.L., Berry S.A., Braverman N. (2020). Treatment of mucopolysaccharidosis type II (Hunter syndrome): a Delphi derived practice resource of the american College of Medical Genetics and Genomics (ACMG). Genet. Med..

[25] Tajima G., Sakura N., Kosuga M., Okuyama T., Kobayashi M. (2013). Effects of idursulfase enzyme replacement therapy for mucopolysaccharidosis type II when started in early infancy: comparison in two siblings. Mol. Genet. Metabolism.

[26] Tomita K., Okamoto S., Seto T., Hamazaki T., So S., Yamamoto T., Tanizawa K., Sonoda H., Sato Y. (2021). Divergent developmental trajectories in two siblings with neuropathic mucopolysaccharidosis type II (Hunter syndrome) receiving conventional and novel enzyme replacement therapies: a case report. JIMD Rep..

[27] Gabrielli O., Clarke L.A., Bruni S., Coppa G.V. (2010). Enzyme-replacement therapy in a 5-month-old boy with attenuated presymptomatic MPS I: 5-year follow-up. Pediatrics.

[28] Dierenfeld A.D., McEntee M.F., Vogler C.A., Vite C.H., Chen A.H., Passage M., Le S., Shah S., Jens J.K., Snella E.M., Kline K.L., Parkes J.D., Ware W.A., Moran L.E., Fales-Williams A.J., Wengert J.A., Whitley R.D., Betts D.M., Boal A.M., Riedesel E.A., Gross W., Ellinwood N.M., Dickson P.I. (2010). Replacing the enzyme α-l-iduronidase at birth ameliorates symptoms in the brain and periphery of dogs with mucopolysaccharidosis type I. Sci. Transl. Med..

[29] Grosse S.D., Lam W.K., Wiggins L.D., Kemper A.R. (2017). Cognitive outcomes and age of detection of severe mucopolysaccharidosis type 1. Genet. Med..

[30] Anderson C., Law J.K., Daniels A., Rice C., Mandell D.S., Hagopian L., Law P.A. (2012). Occurrence and family impact of elopement in children with autism spectrum disorders. Pediatrics.

[31] Guan J., Li G. (2017). Characteristics of unintentional drowning deaths in children with autism spectrum disorder. Inj. Epidemiology.

[32] Cho S.Y., Lee J., Ko A.-R., Kwak M.J., Kim S., Sohn Y.B., Park S.W., Jin D.-K. (2015). Effect of systemic high dose enzyme replacement therapy on the improvement of CNS defects in a mouse model of mucopolysaccharidosis type II. Orphanet J. Rare Dis..

[33] Polito V.A., Abbondante S., Polishchuk R.S., Nusco E., Salvia R., Cosma M.P. (2010). Correction of CNS defects in the MPSII mouse model via systemic enzyme replacement therapy. Hum. Mol. Genet..

[34] Baldo G., Mayer F.Q., Martinelli B.Z., de Carvalho T.G., Meyer F.S., de Oliveira P.G., Meurer L., Tavares A., Matte U., Giugliani R. (2013). Enzyme replacement therapy started at birth improves outcome in difficult-to-treat organs in mucopolysaccharidosis I mice - 1–s2.0-S1096719213000942-main.Pdf. Mol. Genet. Metab..

[35] Dierenfeld A.D., McEntee M.F., Vogler C.A., Vite C.H., Chen A.H., Passage M., Le S., Shah S., Jens J.K., Snella E.M. (2010). Replacing the enzyme α-l-iduronidase at birth ameliorates symptoms in the brain and periphery of dogs with mucopolysaccharidosis type I. Sci. Transl. Medicine.

[36] Vogler C., Levy B., Grubb J., Galvin N., Tan Y., Kakkis E., Pavloff N., Sly W. (2005). Overcoming the blood-brain barrier with high-dose enzyme replacement therapy in murine mucopolysaccharidosis VII. Proc. Natl. Acad. Sci. U. S. A..

[37] Baldo G., Giugliani R., Matte U. (2014). Lysosomal enzymes may cross the blood–brain-barrier by pinocytosis: implications for enzyme replacement therapy. Med. Hypotheses.

[38] Banks W.A. (2006). Are the extracellular pathways a conduit for the delivery of therapeutics to the brain?. Front. Med. Chem..

[39] Zapolnik P., Pyrkosz A. (2021). Gene therapy for mucopolysaccharidosis type II—A review of the current possibilities. Int. J. Mol. Sci..

[40] Poletto E., Baldo G., Gomez-Ospina N. (2020). Genome editing for mucopolysaccharidoses. Int. J. Mol. Sciences.

[41] Leal A.F., Espejo-Mojica A.J., Sánchez O.F., Ramírez C.M., Reyes L.H., Cruz J.C., Alméciga-Díaz C.J. (2020). Lysosomal storage diseases: current therapies and future alternatives. J. Mol. Med..

[42] Hampe C.S., Yund B.D., Orchard P.J., Lund T.C., Wesley J., McIvor R.S. (2021). Differences in MPS I and MPS II disease manifestations. Int. J. Mol. Sciences.

[43] Scarpa M., Orchard P.J., Schulz A., Dickson P.I., Haskins M.E., Escolar M.L., Giugliani R. (2017). Treatment of brain disease in the mucopolysaccharidoses. Mol. Genet. Metabolism.

[44] Shapiro E., Eisengart J. (2021). The natural history of neurocognition in MPS disorders: a review. Mol. Genet. Metabolism.

[45] Barth A.L., de Magalhães T.S., Reis A.B.R., de Oliveira M.L., Scalco F.B., Cavalcanti N.C., Torres D.A., Costa A.A., Bonfim C., Silva D.S. (2017). Early hematopoietic stem cell transplantation in a patient with severe mucopolysaccharidosis II: a 7 years follow-up. Mol. Genet. Metab. Reports.

[46] Eisengart J.B., Rudser K.D., Tolar J., Orchard P.J., Kivisto T., Ziegler R.S., Whitley C.B., Shapiro E.G. (2013). Enzyme replacement is associated with better cognitive outcomes after transplant in hurler syndrome. J. Pediatr..

[47] Eisengart J.B., Jarnes J., Ahmed A., Nestrasil I., Ziegler R., Delaney K., Shapiro E., Whitley C. (2017). Long-term cognitive and somatic outcomes of enzyme replacement therapy in untransplanted Hurler syndrome. Mol. Genet. Metab. Rep..

[48] Tomanin R., Zanetti A., D’Avanzo F., Rampazzo A., Gasparotto N., Parini R., Pascarella A., Concolino D., Procopio E., Fiumara A. (2014). Clinical efficacy of enzyme replacement therapy in paediatric hunter patients, an independent study of 3.5 years. Orphanet J. Rare Dis..

[49] Tan H.-L., Gozal D., Kheirandish-Gozal L. (2013). Obstructive sleep apnea in children: a critical update Nature and science of sleep.

[50] Gagnon K., Baril A.-A., Gagnon J.-F., Fortin M., Décary A., Lafond C., Desautels A., Montplaisir J., Gosselin N. (2014). Cognitive impairment in obstructive sleep apnea. Pathol. Biol..

[51] Froissart R., Da Silva I.M., Maire I. (2007). Mucopolysaccharidosis type II: an update on mutation spectrum. Acta Paediatr..

[52] van der Lee J.H., Morton J., Adams H.R., Clarke L., Eisengart J.B., Escolar M.L., Giugliani R., Harmatz P., Hogan M., Kearney S. (2020). Therapy development for the mucopolysaccharidoses: updated consensus recommendations for neuropsychological endpoints. Mol. Genet. Metab..

[53] Porter K.A., O’Neill C., Drake E., Parker S., Escolar M.L., Montgomery S., Moon W., Worrall C., Peay H.L. (2021). Parent experiences of sanfilippo syndrome impact and unmet treatment needs: a qualitative assessment. Neurol. Therapy.

[54] Shapiro E., Lourenço C.M., Mungan N.O., Muschol N., O’Neill C., Vijayaraghavan S. (2019). Analysis of the caregiver burden associated with sanfilippo syndrome type B: panel recommendations based on qualitative and quantitative data. Orphanet J. Rare Dis..

[55] Wittenberg E., Prosser L.A. (2013). Disutility of illness for caregivers and families: a systematic review of the literature. PharmacoEconomics.

[56] Agency E.M. (2013).

[57] Seo J.-H., Okuyama T., Shapiro E., Fukuhara Y., Kosuga M. (2020). Natural history of cognitive development in neuronopathic mucopolysaccharidosis type II (Hunter syndrome): contribution of genotype to cognitive developmental course. Mol. Genet. Metab. Rep..

[58] Giugliani R., Martins A.M., Okuyama T., Eto Y., Sakai N., Nakamura K., Morimoto H., Minami K., Yamamoto T., Yamaoka M. (2021). Enzyme replacement therapy with pabinafusp alfa for neuronopathic mucopolysaccharidosis II: an integrated analysis of preclinical and clinical data. Int. J. Mol. Sciences.

[59] Kiely B.T., Kohler J.L., Coletti H.Y., Poe M.D., Escolar M.L. (2017). Early disease progression of hurler syndrome. Orphanet J. Rare Dis..

[60] Bailey D.B., Porter K.A., Andrews S.M., Raspa M., Gwaltney A.Y., Peay H.L. (2021). Expert evaluation of strategies to modernize newborn screening in the United States. JAMA Netw. Open.

